# Correlates of unintended pregnancy among currently pregnant married women in Nepal

**DOI:** 10.1186/1472-698X-9-17

**Published:** 2009-08-11

**Authors:** Ramesh Adhikari, Kusol Soonthorndhada, Pramote Prasartkul

**Affiliations:** 1 Geography and Population Department, Mahendra Ratna Campus, Tribhuvan University, Kathmandu, Nepal; 2 Institute for Population and Social Research (IPSR), Mahidol University, Thailand

## Abstract

**Background:**

Women living in every country, irrespective of its development status, have been facing the problem of unintended pregnancy. Unintended pregnancy is an important public health issue in both developing and developed countries because of its negative association with the social and health outcomes for both mothers and children. This study aims to determine the prevalence and the factors influencing unintended pregnancy among currently pregnant married women in Nepal.

**Methods:**

This paper reports on data drawn from Nepal Demographic and Health Survey (NDHS) which is a nationally representative survey. The analysis is restricted to currently pregnant married women at the time of survey. Association between unintended pregnancy and the explanatory variables was assessed in bivariate analysis using Chi-square tests. Logistic regression was used to assess the net effect of several independent variables on unintended pregnancy.

**Results:**

More than two-fifth of the currently pregnant women (41%) reported that their current pregnancy was unintended. The results indicate that age of women, age at first marriage, ideal number of children, religion, exposure to radio and knowledge of family planning methods were key predictors of unintended pregnancy. Experience of unintended pregnancy augments with women's age (odds ratio, 1.11). Similarly, increase in the women's age at first marriage reduces the likelihood of unintended pregnancy (odds ratio, 0.93). Those who were exposed to the radio were less likely (odds ratio, 0.63) to have unintended pregnancy compared to those who were not. Furthermore, those women who had higher level of knowledge about family planning methods were less likely to experience unintended pregnancy (odds ratio, 0.60) compared to those having lower level of knowledge.

**Conclusion:**

One of the important factors contributing to high level of maternal and infant mortality is unintended pregnancy. Programs should aim to reduce unintended pregnancy by focusing on all these identified factors so that infant and maternal mortality and morbidity as well as the need for abortion are decreased and the overall well-being of the family is maintained and enhanced.

## Background

The issue of unintended pregnancy has been essential to demographers seeking to understand fertility, to public health practitioners in preventing unintended childbearing and to both groups in promoting a woman's ability to determine whether and when to have children [1]. Unintended pregnancy can result from contraceptive failure, non-use of contraceptives, and less commonly, rape and it can create serious health consequences for women, children and family [2].

There is very little published literature that focuses on the determinants of unintended pregnancy in developing countries and particularly in Nepal. However, some research studies conducted outside of Nepal have shown the relation between unintended pregnancy and socio-economic and demographic characteristics. Moreover, there is very little known about unintended pregnancy in cultural contexts.

An unintended pregnancy is a pregnancy that is either mistimed (i.e., they occurred earlier than desired) or unwanted (i.e. they occurred when no children, or no more children were desired) at the time of conception [1]. Unintended pregnancy is a potential hazard for every sexually active woman. It is a worldwide problem that affects women, their families, society and nation. A complex set of social and psychological factors puts women at risk of unintended pregnancy. Abortion is a frequent consequence of unintended pregnancy and in the developing countries it can result into serious long-term, negative health effects including infertility and maternal death [2].

Women living in every country, irrespective of the development status, have been facing the problem of unintended pregnancy. Over 100 million acts of sexual intercourse take place each day resulting in around 1 million conceptions, about 50 percent of which are unplanned and about 25 percent are definitely unwanted [3]. The data suggest that approximately 49 percent of all pregnancies in the United States [4], 46 percent in Yamagata, Japan [5], 35 percent in both Iran [6] and Nepal [7] are unintended. Almost all occurred due to non-use of family planning method or contraception failure. About 50 percent of all unintended pregnancies in the United States are due to contraceptive failure [8]. Therefore, unintended pregnancy is an issue that cannot be ignored. Many pregnant women will want or need to end a pregnancy to avoid risks to their lives and health, psychological trauma, and socioeconomic turmoil [9].

International Conference on Population and Development (ICPD) held in Cairo in 1994 and fourth world conference on women held in 1995 in Beijing have emphasized women empowerment as a basic tool for a country's overall development and improving the quality of life of the people[10]. ICPD declared that advancing gender, empowering women and eliminating all kinds of violence against women, and ensuring women's ability to control their own fertility are cornerstones of population and development related programs [11].

A study conducted among college students in Nepal revealed that only about half of the male students (55%) had used condom at the first premarital sexual intercourse [12]. Similarly, the other study showed that 20 percent of rural and 16 percent of urban married women aged 15–49 reported method failure as the reason for their unintended pregnancy [13]. Furthermore, the other research estimated that during the first year of vasectomy, 1.7 percent women could become pregnant [14] which leads to the higher number unintended pregnancies and abortions. Furthermore, a study conducted at 5 major hospitals showed that abortion related hospitalization accounted for 20 percent to 48 percent of the total obstetric and gynecological cases [15]. Despite the legalization of abortion laws (March 2002 onwards) in the country, due to the lack of awareness about the law and facility centers, many women still seek abortion clandestinely and most often they consult unskilled or unqualified health workers, resulting in high rates of abortion related morbidity and mortality [16].

The underlying cause of high prevalence of unintended pregnancy needs further investigation and exploration in order to be better understood and appropriately addressed by reproductive health programs. It is essential to identify the risk factors of unintended pregnancy and to provide services to address those who are at risks. To develop effective strategies for the prevention of unintended pregnancies, it is necessary to understand the factors affecting unintended pregnancies. It is hypothesized that women in the vulnerable group (illiterate, living in the rural area, working on agricultural sector), and who are not exposed to mass media lead to low knowledge of family planning methods and low utilization of the health services which in turn lead to higher unintended pregnancy.

This study aims to determine the prevalence and the factors influencing unintended pregnancy among currently pregnant married women in Nepal. The findings of this study aim to guide reproductive health program planners and policy makers to understand various factors influencing unintended pregnancy and to assist in implementation of the reproductive health program which will decrease unintended pregnancy as well as reduce the risk of maternal and infant morbidity and mortality. Though there are very few studies on unintended pregnancy in Nepal, this type of research which focuses on currently pregnant married women has not yet been undertaken in the country.

## Methods

This paper reports on data drawn from Nepal Demographic and Health Survey (NDHS), 2001 which is a nationally representative sample survey. This cross sectional survey was conducted among married women in the reproductive age (15–49 years). The primary purpose of the NDHS is to generate recent and reliable information on fertility, family planning, infant and child mortality, maternal and child health, and nutrition. The sample for the survey is based on a two-stage, stratified, nationally representative sample of households. At the first stage of sampling, 257 PSUs, 42 in urban areas and 215 in rural areas, were selected using systematic sampling with probability proportional to size method.

Out of 8,726 married women of the reproductive age interviewed, 751 (8.6%) were currently pregnant at the time of the survey. Among those women, 28 respondents were excluded from the analysis due to missing data on the intention status for their current pregnancy. Only currently pregnant women were selected for this study to minimize underreporting of unplanned pregnancies. It also reduces recall bias as it gathers information on the current pregnancy and not on the pregnancy history.

Pregnancy planning is measured by respondents' perceived desire of current pregnancy. The question was "*At any time you became pregnant, did you want to become pregnant then, did you want to wait until later, or did you not want to have any (more) children at all? *The three allowed options were *wanted then (planned), wanted the pregnancy to happen later (mistimed) and did not want at all (unwanted)*. Those respondents who mentioned their current pregnancy is either mistimed or unwanted were merged and consider as 'unintended pregnancy'. Thus, this variable is categorized into two categories: unintended and intended. Women's literacy status is categorized into 2 categories; illiterate and literate. The purpose is to analyze the effect of literacy status on unintended pregnancy (Table 1).

**Table 1:** Operational definitions of variables and their measurements

Variables	Description	Measurement scale
Unintended pregnancy	Type (intendedness) of current pregnancy	*Dichotomous*
		0 = Intended
		1 = Unintended

Age of women	Respondent's current age at time of survey	*Ordinal for bivariate analysis*
		0 = 15–24 years
		1 = 25–34 years
		2 = 35–49 years
		*Interval scale for multivariate*

Ideal number of children	Women's concept or preferences about the number of children	*Ordinal for bivariate analysis*
		1 = One
		2 = Two
		3 = Three and more
		*Interval scale for multivariate*

Parity	Number of children given by the respondents	*Ordinal for bivariate analysis*
		0 = None
		1 = One
		2 = Two
		3 = Three and more
		*Interval scale for multivariate*

Age at first marriage	Respondents' completed age at the time of marriage	*Ordinal for bivariate analysis*
		0 = Less than 16 years
		1 = 16 years and more
		*Interval scale for multivariate*

Women's education	Literacy status of women	*Ordinal*
		0 = No education/illiterate
		1 = Literate

Women's occupation	Types of women's work	*Nominal*
		0 = Not working/agriculture
		1 = Non-agriculture

Place of residence	Types of place of residence of the respondent	*Dichotomous*
		0 = Urban
		1 = Rural

Radio exposure	Listen to radio every day	*Dichotomous*
		0 = No
		1 = Yes

TV exposure	Watch television at least once a week	*Dichotomous*
		0 = No
		1 = Yes

Travel time to the nearest family planning sources	Travel time needed to reach the nearest family planning sources from her residence	*Ordinal*
		0 = Less than 30 minutes
		1 = 30–60 minutes
		2 = More than 1 hour
		3 = No response/don't know

Family planning field worker's visit	Women who are visited by family planning program's worker in the last 12 months	*Dichotomous*
		0 = Not visited
		1 = Visited

Religion	Women's religion	*Dichotomous*
		0 = Non-Hindu
		1 = Hindu

Woman's autonomy	Autonomy on own health care and how to spend own earned money	*Nominal*
		0 = No autonomy
		1 = Some autonomy

Knowledge about family planning methods	Knowledge score of different family Planning method	*Ordinal*
		0 = Lower knowledge
		1 = Higher knowledge

Ever use of family planning method	Respondents who had ever use of any contraceptive or not in the past	*Dichotomous*
		0 = Never used
		1 = Ever used

Association between unintended pregnancy and the explanatory variables was assessed in bivariate analysis using Chi-square tests. Logistic regression was used to assess the net effect of several independent variables on unintended pregnancy. Before the multivariate analysis, multicollinearity between the variables was assessed and the least important variables were removed from the logistic model. Statistical Package for Social Science (SPSS) was used for analysis.

## Results

Among the surveyed married women of reproductive age, less than one in ten respondents (8.6% out of 8,726) was currently pregnant at the time of the survey. Among these currently pregnant respondents, about one-fifth mentioned that they wanted their current pregnancy later (mistimed; 21%) and the other one-fifth reported that they did not want their current pregnancy at all (unwanted; 20%). Conclusively, more than two-fifth of the currently pregnant married women (41%) reported their current pregnancies were unintended.

When stratifying the women in different characteristics, it was found that the percentage of women who have experienced current pregnancy as unintended varied by different settings. More than two-fifth illiterate women (44%), women who had no job or worked in agricultural sector (42%), and resided in rural area (42%) had significantly higher incidence of unintended pregnancy compared to their counterparts. In terms of religion, more than half of non-Hindu women (52%) while only about two-fifth of Hindu women (39%) had reported their current pregnancy as unintended (Table 2).

**Table 2:** Pregnancy intention by selected characteristics

		Experience of unintended pregnancy (%)	Total Number
**Demographic characteristics**			

Age group***	15–24	31.3	415
	25–34	48.4	247
	35–49	76.5	61

**Ideal number of children**#	1–2 children	39.2	404
	Three or more	44.4	303

Total children ever born***	None	20.7	195
	One	28.8	184
	Two	48.2	122
	Three or more	64.9	222

Age at first marriage**	Less than 16 years	46.2	339
	16 year or more	36.3	384

**Socio-economic characteristics**			

Literacy status**	Illiterate	44.4	486
	Literate	34.0	237

**Occupation**	Not working/agriculture	41.8	671
	Non agriculture	30.4	52

**Place of residence**	Rural	41.7	673
	Urban	31.7	50

**Access to health information/services**			

Listens to radio **	No	45.3	469
	Yes	33.0	254

**Watches television**	No	42.5	583
	Yes	34.6	140

Travel time to nearest family planning center ##**	Up to 30 minutes	38.0	363
	31–60 minutes	45.0	167
	More than one hour	54.1	91

Family planning worker visit*	Not visited	39.8	663
	Visited	54.0	60

**Socio-cultural factors**			

Religion**	Non-Hindu	52.2	112
	Hindu	38.9	611

Women autonomy*	No autonomy	38.7	571
	Some autonomy	49.7	152

**Knowledge and practice of family planning method**			

Knowledge about family planning method**	Lower	46.4	411
	Higher	33.8	312

**Ever use of family planning method**	Never use	39.4	518
	Ever use	44.9	205

	**Total**	**41.0**	**723**

*Note*: * = p < .05, ** = p < .01 *** = p < .001, # Those respondents who didn't know the sources of FP methods are excluded, ## Travel time is only for those who knew the sources of FP

As expected, the percentage of women reporting unintended pregnancies increased with age (31% of the women aged less than 25 years to 77% of the women aged 35 and above years). Similarly, women with higher birth order reported significantly higher rate of unintended pregnancy. Furthermore, women who got married at early age (before 16 years) had significantly higher rate of unintended pregnancy (46%) compared to those who got married at 16 years or later (36%).

The result shows that the exposure to mass media is significantly negatively associated with the level of unintended pregnancy. For instance, only about one third of the respondents who were exposed to the radio have reported their current pregnancy as unintended (33%) while the proportion was more than two-fifth (45%) for those who were not exposed to radio. Similarly, access to health services is negatively associated with the proportion of unintended pregnancy. Those respondents who resided near the family planning sources (less than 30 minutes travel distance) reported significantly much lower (38%) experienced unintended pregnancy compared to those who resided far (more than 1 hour travel distance) from the family planning sources (54%). Likewise, the study found that higher the level of knowledge of family planning methods, the lower the percentage of women reporting the current pregnancy as unintended (34%). Against expectation, those respondents who were visited by family planning workers in the last 12 months had higher level of unintended pregnancy (54%) compared to those who were not visited by family planning worker (40%). Similarly, women who have some autonomy had significantly higher level of unintended pregnancy (50%) than those who have no autonomy (39%) (Table 2).

Binary logistic regression model was used to assess the net effect of each of the independent variables on the dependent variable, while controlling for the other variables in the model. Three models had been used in the analysis. The first model contained the individual factors such as demographic characteristics, socio-economic factors, and access to health information/services. In the second model, socio-cultural factors were added. In the third model, intervening variables such as knowledge and ever use of family planning methods were added and the effect of intervening variables and independent variables on unintended pregnancy was observed. After assessing multicollinearity in the variables, it was found that the variables 'age of women' and 'number of children ever born' were highly correlated. So the variable 'children ever born' was not entered in the logistic regression model.

In the first model, age of the women has positive and statistically significant impact on unintended pregnancy. On the other hand, ideal numbers of children, age at first marriage and exposure to the radio have negative and statistically significant effect with unintended pregnancy. The results indicate that with an increase in women's age, the odds of women experiencing unintended pregnancy also increases (OR, 1.12) by keeping other individual variables constant in the model. In terms of ideal number of children, the likelihood of reporting unintended pregnancy decreases (OR, 0.76) with an increase in the ideal number of children. Similarly, increase in age at first marriage reduces the likelihood of unintended pregnancy among women (OR, 0.94). Regarding radio exposure, those who were exposed to the radio were less likely to have unintended pregnancy (OR, 0.60) compared to those who were not exposed.

All these four variables retained their significance even after inclusion of socio-cultural factors (religion and women's autonomy) in the model 2. The reduction on the odds ratio of the variables such as age, ideal number of children, age at first marriage, radio exposure after inclusion of socio-cultural variables indicated that the socio-cultural factors were also important predictors of unintended pregnancy. Model 2 further explained that Hindu women were less likely to have experienced unintended pregnancy (OR = 0.48) compared to other religion keeping all other variables constant in the model.

Model 3 presents the final results after adding intervening variables in model 2. Even after inclusion of the knowledge and ever used of family planning methods variables in model 3, the four individual and one socio-cultural variable were still statistically significant. Furthermore, out of two intervening variables, knowledge about family planning methods had statistically significant effect (OR = 0.60) on experience of unintended pregnancy. Those women who had higher level (more than average score) of knowledge about family planning methods are less likely (OR = 0.60) to experience unintended pregnancy compared to those who have lower level of knowledge (less than average score) about family planning methods (Table 3).

**Table 3:** Estimated odds ratios for having unintended pregnancy among currently pregnant married women by selected predictors

		Odds ratios		
		
		Model (I)	Model (II)	Model (III)
**Demographic characteristics**	**Age **(in years)	1.112***	1.106***	1.105***
	
	**Ideal number of children **(number)	0.761*	0.751*	0.725**
	
	**Age at first marriage **(in years)	0.937**	0.926**	0.929*

**Socio-economic characteristics**	**Literacy**			
	Illiterate (ref.)			
	Literate	1.221	1.212	1.336
	
	**Occupation**			
	Not working/agriculture (ref.)			
	Non-agriculture	0.708	0.587	0.580
	
	**Place of residence**			
	Urban (ref.)			
	Rural	0.981	0.963	0.984

**Access to health information/services**	**Listens to radio **			
	No (ref.)			
	Yes	0.603**	0.583**	0.628*
	
	**Watches television **			
	No (ref.)			
	Yes	0.930	0.954	0.959
	
	**FP worker visit**			
	Not Visited (ref.)			
	Visited	1.385	1.199	1.274
	
	**Travel time to nearest FP source**			
	Up to 30 minutes (ref.)			
	31–60 minutes	1.200	1.159	1.110
	More than one hour	1.549	1.460	1.344
	No response	0.665	0.699	0.607

**Socio-cultural factors**	**Religion**			
	Non-Hindu (ref.)			
	Hindu	-	0.482**	0.468**
	
	**Women autonomy**			
	No autonomy (ref.)			
	Some autonomy	-	1.305	1.374

**Knowledge and practice of FP**	**Knowledge of FP**			
	Lower (ref.)			
	Higher	-	-	0.600**
	
	**Ever use of FP**			
	No (ref.)			
	Yes	-	-	0.994

	***-2 log likelihood***	***868.1***	***852.0***	***844.9***

	***Cox & Snell R square***	***0.102***	***0.122***	***0.131***

Note * = p < .05, ** = p < .01 *** = p < .001, ref = reference category

## Discussion

This study has attempted to investigate the influencing factors such as demographic, socioeconomic, socio-cultural, access to health information/services and knowledge and ever use of family planning methods on unintended pregnancy. Present study showed that unintended pregnancy is common among Nepalese women. It indicates higher demand for family planning program. The result of this study suggests that all women, regardless of age, socioeconomic, or socio-cultural status, would benefit from increased efforts to ensure that pregnancies are intended.

The bivariate analysis showed that the variables such as age, total children ever born, age at first marriage, literacy status, radio exposure, travel time to the nearest family planning source, family planning workers' visit, religion, women's autonomy and knowledge about family planning methods are important in explaining unintended pregnancy. The multivariate analysis supported some of the findings of the bivariate analysis and indicated a different pattern of effect for few other variables. In the multivariate analysis, age of women, ideal number of children, age at first marriage, radio exposure, religion and knowledge about family planning methods were found to have statistically significant influence on unintended pregnancy.

This study has shown that the higher the age of women, the higher the probability of having current pregnancy as unintended. It is similar to the study conducted in currently married pregnant women in Iran [6] and all women of reproductive age in Nigeria [17].

A contradictory result was observed from the logistic regression regarding the association of ideal number of children on an unintended pregnancy. In the multivariate analysis, ideal number of children was negatively associated with unintended pregnancy indicating that those women who desired more children were less likely to experience unintended pregnancy. One reason could be more people (93%) live in rural areas and rural women perceive greater benefit from having more children. Hence our sample reflected that the decline in desired family size in Nepal resulted in increased exposure to the risk of having unintended pregnancy.

Like the study in Japan [5], we found significant negative relationship between age at first marriage and unintended pregnancy in Nepal. One of the reasons could be that early marriage leads to earlier initiation of sexual intercourse, which exposes women to an extended period when they are at risk of getting pregnant and is thus related to a higher likelihood of experiencing unintended pregnancy. The other reason could be that the women who married early may have limited access to services or may experience particular difficulty in practicing contraception.

The multivariate results showed that those who have had regular access to mass media (radio) were less likely to report unintended pregnancy compared to those who have not. It means mass media has played an important role in reducing unintended pregnancy because it gives wider range of knowledge [18,19] and leads to adopt contraception and sensitizes couple about the family norms so that they have low parity and low unintended pregnancy [20,21].

Unintended pregnancy was more common in non-Hindu women compared to Hindu women. One of the reasons could be that Hindu women are likely to accept pregnancy as "Given by God" or "Treasure of the Family". The other reason might be due to considerable proportion (38%) of Muslim women included in non-Hindu category. Islam restricts women's activities in ways that other religions do not [22].

We hypothesized that women who have higher knowledge about family planning methods (more than average) are less likely to experience unintended pregnancy. Our result supports the hypothesis that if a woman has higher knowledge of family planning methods, she is more likely to be aware of the benefits of those methods which in turn will motivate her to use the family planning methods and be less likely to have unintended pregnancy. The similar result is found in Ecuador as well [23].

In this study, there was no significant association between the experience of unintended pregnancy and women's education as in Japan [5], and occupation like the study found in Iran [6]. In Japan, most of the women are educated and they prefer not to have children or to have fewer children compared to other Asian countries. So there is no significant difference in the experience of unintended pregnancy among different educational levels of Japanese women. In case of Nepal, the literacy rate of women is very low and a large number of women do not have more than primary education and other social cultural factors strongly influence the intended pregnancy status; hence education is statistically not significant. However, it should not be concluded that education is not significantly related to intended pregnancy status and thus we should not ignore the importance of education for the better life of women.

Similarly, contrary to the hypothesis, the present study found that women's autonomy has no significant impact on unintended pregnancy. In this study, women's autonomy was measured from the final say on their 'own health care' and 'spending their own earned money'. This is because in a patriarchal society, women are often given less opportunity to be self-supporting and have to depend on the male partners/relatives for their survival [24] and the possibility that women who earned cash are associated with households of low economic status and the job itself was low status jobs.

Although statistically not significant, women who had exposure to Television and lived near health facilities had lower chances of unintended pregnancy than women in the comparison group. Ever use of family planning method has significant relationship with intended pregnancy status of women in many literatures. However, the result from this study is not similar to those findings. Some of the reasons identified were the complexity of using contraceptive or lack of methods choice and financial barriers hindering effective use of contraceptive methods. It was seen that the individual or community perception about contraception is an important factor, which affects contraceptive use. Similarly, misconception leads to discontinuation and decreased use of contraception and increases the level of unintended pregnancy [10]. Thus it can be argued that misconception about family planning methods exist among Nepalese women. High family planning method failure among married women in the reproductive age is also prevalent in Nepal [13]. However it does not imply that contraceptive use is not an important determinant of unintended pregnancy among married pregnant women in Nepal, it rather reflects the situation that the variable ever use of family planning methods acts indirectly on unintended pregnancy in this study.

The concept of "intended ness of pregnancy" is complex and it would probably be better to treat it as a continuous rather than a bicategorical variable [25]. Women are often ambivalent about their intention to become pregnant or not. Nonetheless, measures of unintended pregnancy that use the intended/unintended dichotomy remain valuable because they allow us to assess trends over time and differences among population subgroups [26]. It has been shown that the perception of intended ness of pregnancy varies during the gestational period and after the delivery [27]. The use of a measure of mistimed pregnancies may be especially problematic, since a birth can be mistimed by a short amount of time or a longer period of time, each possibly having different implications [1,28]. Furthermore, many studies compare only intended pregnancies to unintended pregnancies, but do not examine mistimed and unwanted pregnancies separately, even though studies that do separate unwanted from mistimed pregnancies have found many differences in the mother's interpretation of pregnancy intention and the outcomes associated with it [1,2,4-6,26,29-32]. Moreover, if we take children born in the preceding five years or life time, that information may in fact underestimate unplanned childbearing since women may rationalize unplanned births and declare them as planned once they occur. The data used in this paper recorded the intendedness of current pregnancy among the currently pregnant women. It also minimizes underreporting of unintended pregnancy as well as reduces recall bias. In that sense, our study must be less biased than other studies that interview women at different times after delivery.

There are some limitations to interpret the results of this study. First, as pointed out previously, we restricted our subjects to only currently pregnant married women at the time of survey, so obtained prevalence of women with experience of unintended pregnancy should not be generalized to the general population in Nepal. The main objectives of this study are to determine the prevalence and examine the factors influencing unintended pregnancy among currently pregnant married women in Nepal. Thus we intentionally selected a group of women who were currently pregnant during the period of survey, though risk factors of mistimed and unwanted pregnancy is not same, Second, because a cross sectional design of the study and all of the items analyzed in the logistic regression analysis were information at the time of survey, the analysis can only provide evidence of statistical association between those items and the experience of unintended pregnancy and cannot show the cause-effect relationships.

## Conclusion

In conclusion, no single factor accounted for the high rates of unintended pregnancy; many factors contributed in this regard. Among them, this study has found that age of women, perceived ideal number of children, women's age at first marriage, radio exposure, religion and knowledge of family planning methods are strong predictors of unintended pregnancy. In short, it can be concluded that program should aim to reduce unintended pregnancy by focusing on all these identified factors so that infant and maternal mortality and morbidity as well as the need for abortion is decreased and the overall well-being of the family is maintained and enhanced.

## Competing interests

The authors declare that they have no competing interests.

## Authors' contributions

RA conducted data analysis, interpretation of the data and drafted the manuscript. KS and PP provided comments, read and approved the final manuscript. All authors read and approved the final version of the manuscsript.

## Pre-publication history

The pre-publication history for this paper can be accessed here:

http://www.biomedcentral.com/1472-698X/9/17/prepub
